# Development of zebrafish paired and median fin musculature: basis for comparative, developmental, and macroevolutionary studies

**DOI:** 10.1038/s41598-018-32567-z

**Published:** 2018-09-21

**Authors:** Natalia Siomava, Fedor Shkil, Elena Voronezhskaya, Rui Diogo

**Affiliations:** 1 Department of Anatomy, Howard University College of Medicine, 520 W Street NW, 20059 Washington, DC USA; 2 Koltzov Institute of Developmental Biology, Russian Academy of Sciences, ul. Vavilova 26, 119334 Moskva, Russia; 3 Severtsov Institute of Ecology and Evolution, Russian Academy of Sciences, pr. Leninskii 33, Moscow, 119071 Russia

## Abstract

The model organism *Dario rerio* (zebrafish) is widely used in evo-devo and comparative studies. Nevertheless, little is known about the development and differentiation of the appendicular musculature in this fish. In this study, we examined the development of the muscles of all five zebrafish fin types (pectoral, pelvic, anal, dorsal and caudal). We describe the development of the muscles of these fins, including some muscles that were never mentioned in the literature, such as the *interhypurales* of the caudal fin. Interestingly, these caudal muscles are present in early stages but absent in adult zebrafishes. We also compare various stages of zebrafish fin muscle development with the configuration found in other extant fishes, including non-teleostean actinopterygians as well as cartilaginous fishes. The present work thus provides a basis for future developmental, comparative, evolutionary and evo-devo studies and emphasizes the importance of developmental works on muscles for a more comprehensive understanding of the origin, development and evolution of the appendicular appendages of vertebrate animals.

## Introduction

*Danio rerio* (zebrafish) (Teleostei; Actynopterygii) is a model organism that is widely used in various fields of biological research. A significant part of evolutionary studies use this model for evo-devo comparisons with different vertebrate taxa and for general discussions on paired fin-limb transitions that occurred during the origin of the tetrapod lineage^1–5^. However, most of such studies are based on gene expression changes and anatomical comparisons of the skeleton, not including details about soft tissues such as muscles^2,3^. In fact, there is little information available about the adult musculature of the five fin types (pectoral, pelvic, dorsal, anal, and caudal) of the zebrafish^6–9^. In order to replenish our knowledge and understanding of the muscle anatomy of different types of fins in the zebrafish, we recently described the musculature of these fins in adult specimens^10^. The results of that work supported the idea that the zebrafish is an appropriate model organism for studies on the appendicular musculature of teleosts and even of actinopterygians as a whole in at least the case of the paired fins. That recent work paved the way to establish homologies between the appendicular structures of zebrafish and other vertebrates and for discussions on evolutionary trends within these animals^10^.

Surprisingly, despite the common use of the zebrafish as a model organism for Evo-Devo discussions on both paired and median appendages, almost nothing is known about the development and differentiation of the fin musculature in these fishes. Grandel and Schulte-Merker’s 1998^11^ work was mainly focused on the apical epidermis and endoskeletal morphogenesis; myogenesis was only briefly reviewed. Thorsen and Hale published a study describing the development of the musculature of the zebrafish pectoral fin^9^, but they did not refer to some pectoral muscles, such as the arrector-3^10^. Patterson and colleagues studied the growth of the pectoral fin and trunk musculature and looked at different fiber types that constitute the undifferentiated abductor and adductor muscle masses^12^, but the differentiation of those muscle masses and development of individual pectoral fin muscles were not the focus of that study. One of the aims of the present work is therefore to provide developmental data for all the muscles of the pectoral appendage of the zebrafish. Moreover, we compare our results with previous observations in order to provide the most updated and complete information about these muscles.

Studies on the zebrafish pelvic fins mostly focus on the earlier developmental stages when those fins are formed. For instance, Cole *et al*. provided a general discussion on the early development, as well as evolution, of the musculature of the pelvic appendage^13^, focusing mainly on developmental mechanisms and migration of muscle precursors. Other studies focused on the genetic mechanisms involved in the positioning^14^, induction and differentiation of the pelvic fin buds in various fish families^15–17^. Instead, in the present paper, we are mainly interested on the development of individual pelvic muscles until they display a configuration similar to that of adults. More strikingly, the development of the musculature of the median (anal, dorsal and caudal) fins of the zebrafish has never been studied, even in very earlier stages.

In order to tackle this scarcity of information on the development of the zebrafish appendicular musculature, in this paper we describe the ontogeny of each muscle in each fin, i.e. of *each* appendicular muscle, from the time it first becomes visible until when it displays a configuration basically similar to that of the adult stage. We then compare various stages of zebrafish fin muscle development with the configuration found in other extant fishes, thus providing a basis for future developmental, comparative, evolutionary and evo-devo studies on the appendicular musculature of gnathostomes.

## Materials and Methods

Fertilized eggs were obtained by natural mating wild-type (AB) zebrafishes (*Danio rerio*). Embryos and larvae were kept at 28,5 °C ± 0.2 °C in a 25 L plastic aquaria on 14 h light/10 h dark cycle. Once in three days, 1/5 of the total water volume was renovated in the tank. The newly hatched larvae were fed on TertraMin baby (Tetra, Germany) and NovoTom artemia (JBL, Germany). A week after the foraging onset, the diet was changed to TertraMin Junior (Tetra, Germany) and Artemia Salina shrimps (Artemia Koral, Russia). Every day, 3 to 5 fishes were euthanized with the overdose of anesthetic MS-222 (Sigma-Aldrich, Germany) and fixed for 12–14 h at 4 °C in 4% paraformaldehyde (Panreac, Spain) in 0.01 M phosphate buffer saline (PBS pH 7.4, Gibco, Germany) supplemented with 10 μl of 0.5% Alizarin Red (Sigma, USA) diluted in water per 1 ml of the fixation solution. After fixation, samples were washed three times in PBS and stored in PBS with antimicrobial agent 0.1% Thymol (Fluka Analytical, USA) at 4 °C.

Before the phalloidin staining, samples were washed in fresh PBS, transferred in PBS-TX (5% Triton X-100 in PBS), and incubated for 72 h at 10 °C. During incubation, the PBS-TX buffer was refreshed three to four times. The staining was performed in phalloidin-Alexa 488 (Invitrogen, Molecular Probes, A 12379, USA) diluted 1:500 in PBS-TX during 24 h. Stained samples were washed 3 times in PBS ans mounted in 85% fructose in PBS via gradual increase of the fructose concentration in the solution (30%, 50%, and 70%). Slides were stored at room temperature until examination.

The developmental stage of the specimens was estimated by the length of the body. The notochord length (NL) of preflexion fish larvae was measured from the anterior end of the upper jaw to posterior tip of notochord^18^. In fishes with a bending posterior tip of the notochord, the standard length (SL) was measured from the anterior end of the upper jaw to the posterior end of the hypurals^18,19^. The length of every fish was measured under the stereomicroscope Olympus SZX7 with an ocular micrometer. Each developmental stage was examined as whole-mounts under the laser scanning microscope Leica TCS SP5 (Leica, Germany) equipped with the Ar 488 laser for the phalloidin visualization and the DPSS 561 laser for the alizarin visualization. The laser intensity and wavelength-filter configuration were set up the capture all details. When necessary, fish larvae were scanned from both sides. For each larva, 150–250 optical sections with thickness 1.0–1.5 μm were taken and processed with Leica LAS AF (Leica, Germany) and inspected with the ImageJ (NIH, USA) software. In total, approximately 100 specimens were inspected. We selected only a few specimens to be shown in the figures and referred in detail in the text. This is because we did not see apparent cases of intraspecific variability concerning the time of ontogenetic appearance of the muscle features analyzed in this work. Because developmental time of the pectoral fins is relatively long, 23 specimens representing 18 different stages were recorded. For the pelvic, dorsal and anal fins - which develop faster - eight specimens were recorded for each type of fin. As the developmental patterns of muscle development in the caudal fin was previously unknown, we inspected 28 specimens representing 23 stages of development of this fin. In some of the 100 studied specimens, the development of the muscles of more than one fin was analyzed in detail.

Series of optical sections containing relevant structures were projected into a single image and exported as TIFF files. Due to a variant number of optical sections required for every image, the brightness and contrast were adjusted with ImageJ for each panel separately. The nomenclature of the developing zebrafish appendicular muscles follows that of adult zebrafishes in Siomava and Diogo^10^.

All zebrafish experiments were approved by ethics committees of the Russian Academy of Sciences. All procedures were carried out according to the guidelines and following the laws and ethics of Russian Federation and USA. All data generated and analyzed during this study are available from the corresponding author on reasonable request.

## Results

**Figure 1:**
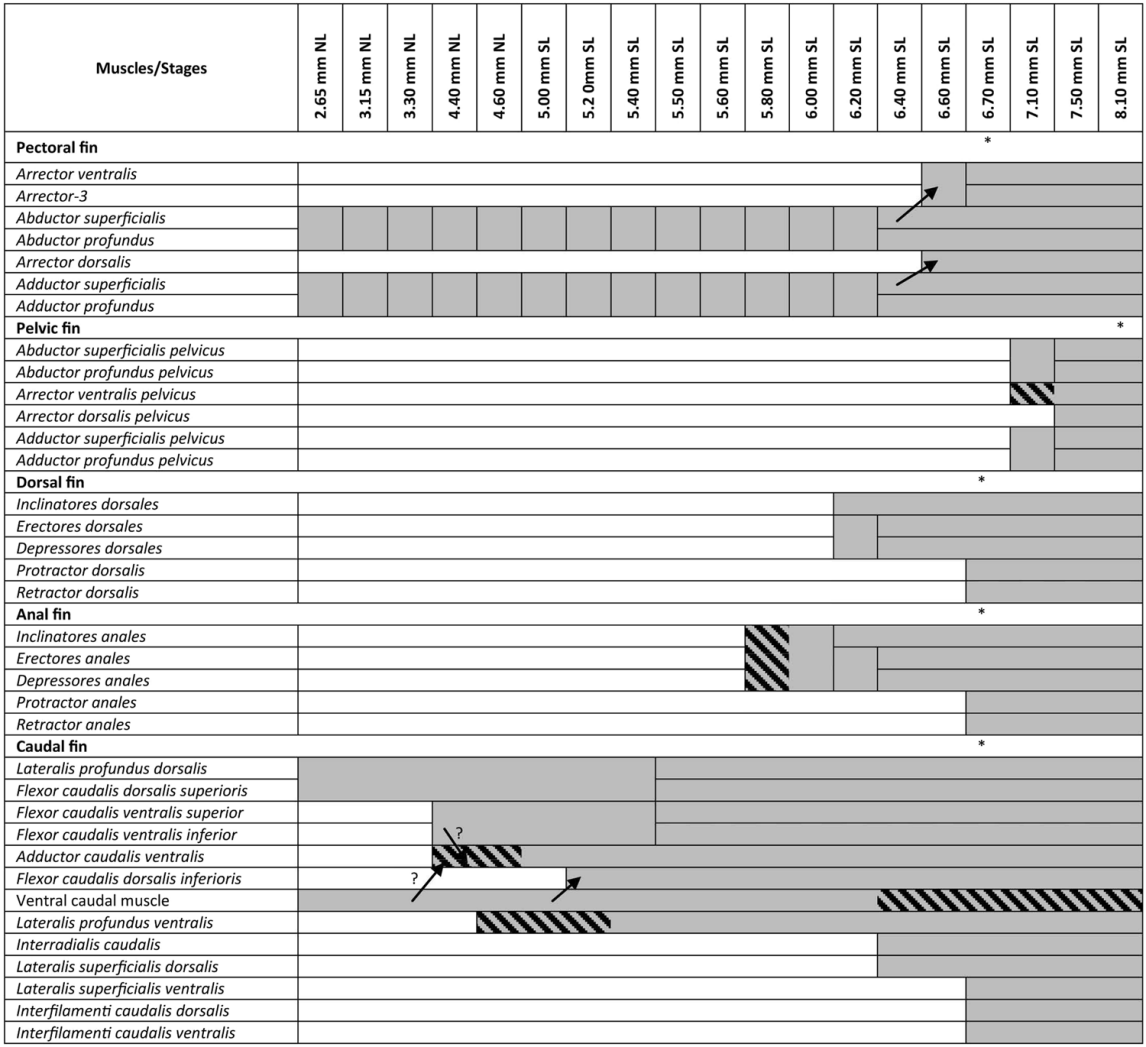
Diagram of the development of appendicular muscles in the zebrafish. Arrows indicate development from another muscle. Stars mark the stage when the adult muscle configuration is achieved. “?” refers to the question of whether the ventral caudal muscle of flexor caudalis ventralis contribute fibers to the adductor caudalis ventralis. Shaded cells show stages when muscles have no attachment to fin rays.

The results are briefly summarized in Fig. 1.

### Caudal fin

**Figure 2:**
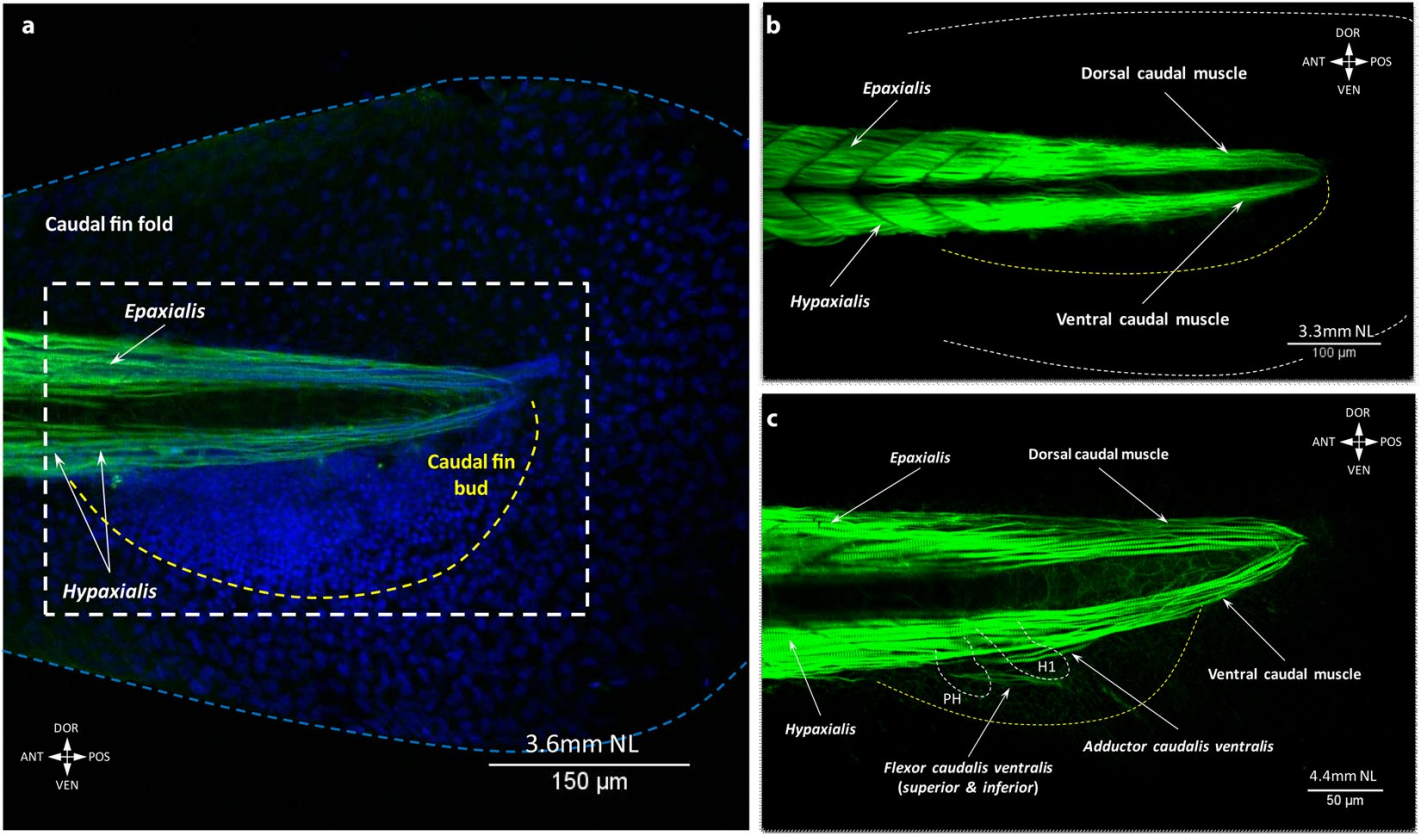
Early development of the caudal fin musculature in the zebrafish. The tip of the tail is shown with the caudal fin fold (dashed blue line) and mesenchyme concentrated in the caudal fin bud (dashed yellow line) (**a**). The white dashed line schematically outlines the area of interest shown in other figures. At 3.3 mm NL, two muscles are present in the caudal fin (**b**). Ventral caudal muscles develop before dorsal muscles. At 4.4 mm NL, PH – parhypural, H1 – hypural 1, and first fibers of ventral caudal muscles can be seen (**c**).

The caudal fin is the first fin to develop (Fig. 1). It appears as a continuation of the zebrafish postcranial axial skeleton and is surrounded by the caudal fin fold with the mesenchyme condensation ventrally, where the first caudal muscles and bones will later develop (Fig. 2a). At stage 2.95 mm NL, it is already associated with muscles. By 3.30 mm NL inclusive, two muscle masses - dorsal and ventral caudal muscles - are continuous with the trunk muscles (epaxialis and hypaxialis, respectively). They expand posteriorly to the tip of the tail (Fig. 2b). Even though there is no clear border between these early caudal muscles and the trunk muscles, they can be distinguished from myotomes by the secondary absence of segmental boundaries.

**Figure 3:**
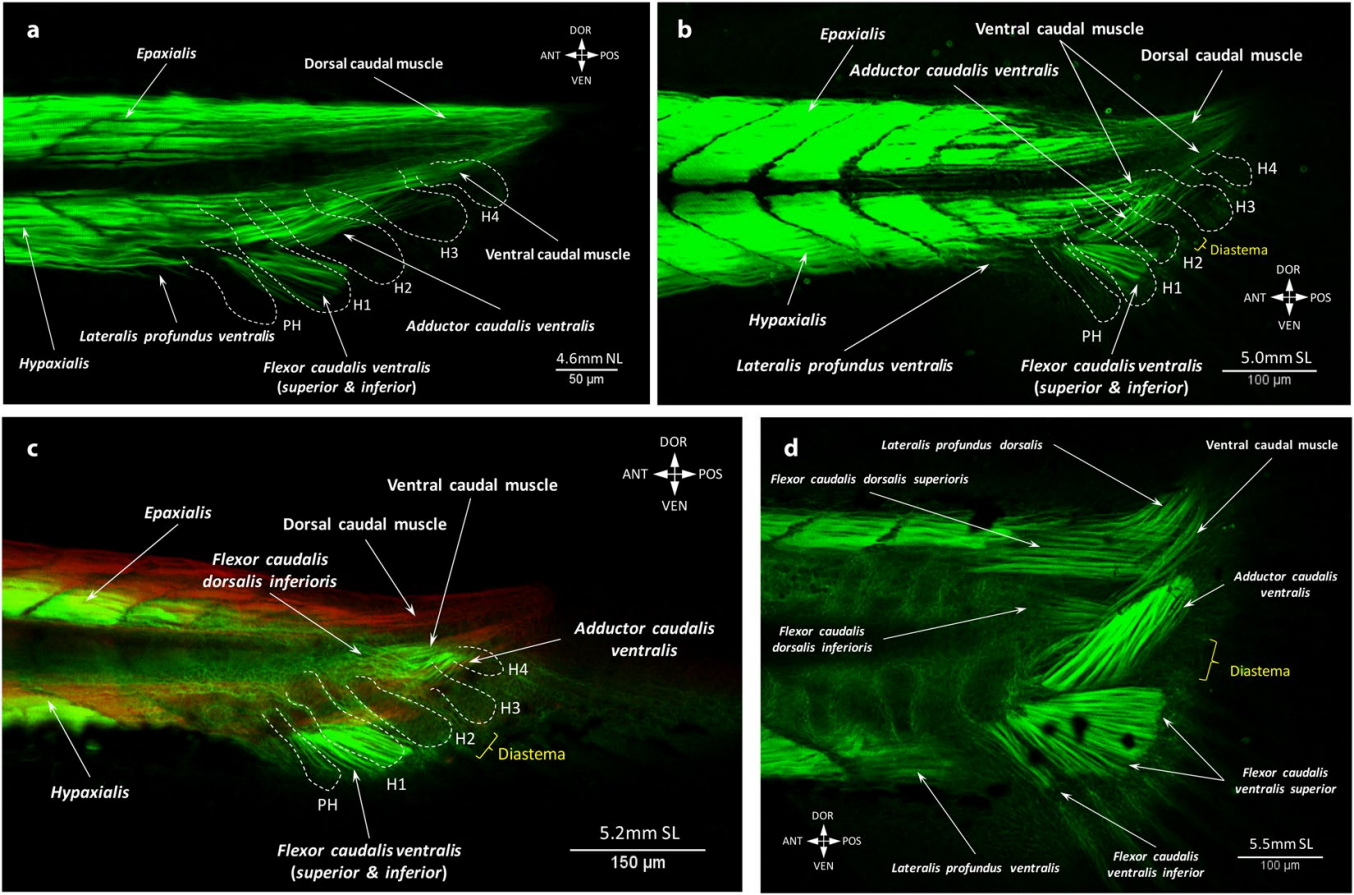
Development of the deep dorsal caudal fin muscles in the zebrafish. At 4.6 mm NL, ventral caudal muscles grow towards the caudal fin rays are present. (**a**) By 5.0 mm SL, ventral caudal muscles reach the caudal fin rays (**b**) and development of the deep dorsal fin muscles starts (**c**,**d**). The flexor caudalis dorsalis inferioris can be seen at 5.2 mm SL (**c**) and the flexor caudalis dorsalis superioris appears at 5.5 mm SL (**d**). Note: different colors in (**c**) do not mean different staining, but were used to contrast the flexor caudalis dorsalis inferioris and the dorsal caudal muscle lodging in different layers. The ventral caudal muscle is still attached to the dorsal caudal fin rays and the flexor caudalis ventralis splits into the superior and inferior portions (**d**). Hypurals and parhypural are outlined with the white dashed line (PH – parhypural, H1 – hypural 1, H2 – hypural 2, H3 – hypural 3, H4 – hypural 4, H5 – hypural 5).

At 4.4 mm NL, three new muscles appear on the ventral side of the caudal fin (Figs 1c and 2a). Myofibrils of the adductor caudalis ventralis and flexor caudalis ventralis, which at this time included both the flexor caudalis ventralis superior and inferior, start bifurcating from the ventral caudal muscle (Fig. 2c, Supplementary Fig. S1). The flexor caudalis ventralis runs ventrally towards the caudal fin fold. The adductor caudalis ventralis mainly follows the direction of the ventral caudal muscle but has shorter fibers that stop halfway to the tail tip. Several short muscle fibers of the lateralis profundus ventralis start separating from the hypaxialis at this stage. The adductor caudalis ventralis becomes more segregated but still keeps the direction of the ventral caudal muscle (Fig. 3a, Supplementary Fig. S2). The flexor caudalis ventralis acquires more fibers and short fibers of the lateralis profundus ventralis extend from the hypaxialis ventrally. Even later in development, the lateralis profundus ventralis does not separate from myomeres. In adult specimens, this latter muscle remains rooted into several posterior caudal myomeres^8,10^. By 5.0 mm SL, when the notochord starts bending dorsally, both the adductor caudalis ventralis and flexor caudalis ventralis substantially increase in size (Fig. 3b). The flexor caudalis ventralis is attached to the ventral rays. The adductor caudalis ventralis becomes more separated from the ventral caudal muscle and changes the direction towards the dorsal fin rays. At this stage, fibers of the lateralis profundus ventralis do not reach the caudal rays.

**Figure 4:**
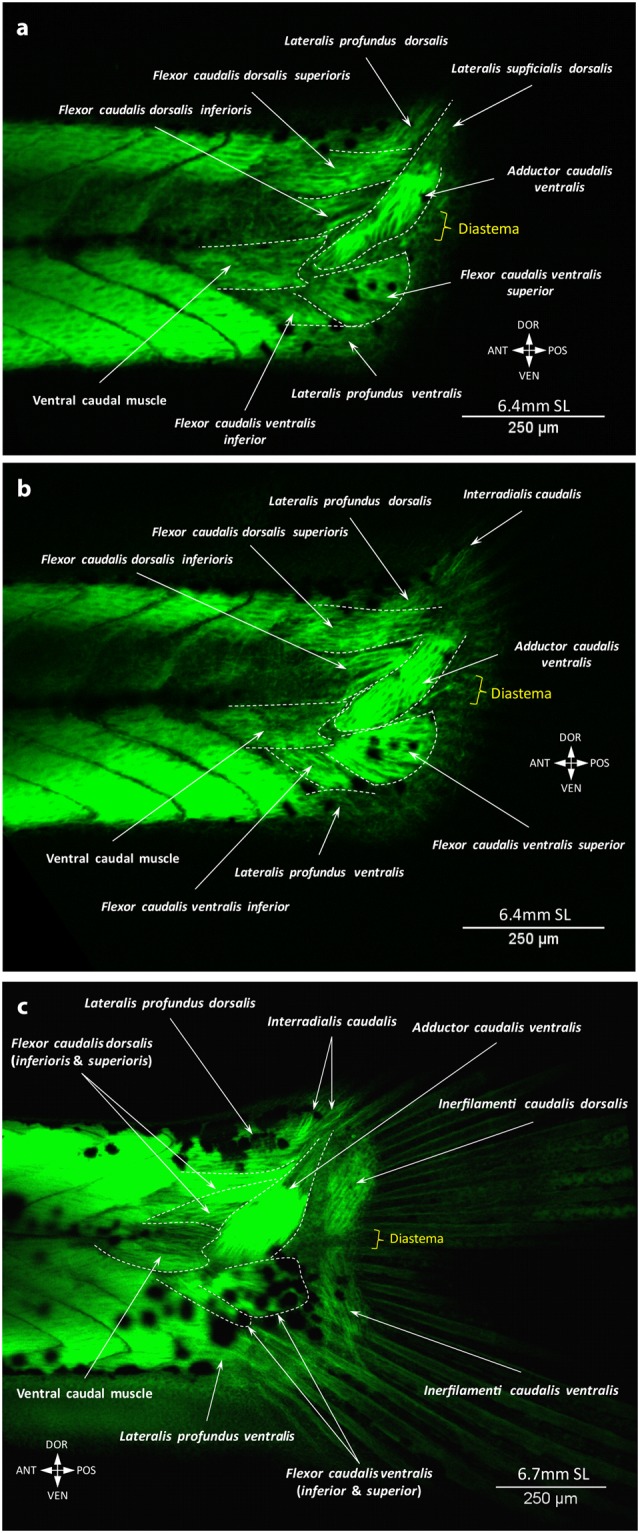
Late development of the caudal fin muscles in the zebrafish. At 6.4 mm SL, the lateralis superficialis dorsalis (**a**) and interradialis caudalis (**b**) are formed. The ventral caudal muscle is shifted backward and attached to the proximal caudal bones and vertebrae. At 6.7 mm SL, the inerfilamenti caudalis dorsalis and ventralis are formed and the development of the caudal musculature is complete (**c**).

**Figure 5:**
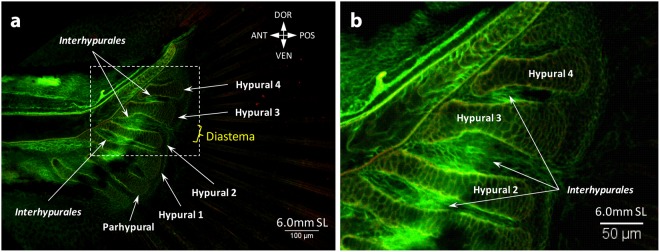
Deep interhypural fibers at 6.0 mm SL. Interhypurales were present between hypurals of the caudal fin from 5.00 mm SL to 7.1 mm SL. Panel (b) shows a high magnification of the rectangle outlined in the panel (a).

At stage 5.2 mm SL, the flexor caudalis dorsalis inferioris can be seen for the first time, arising deeply from the dorsal side of the ventral caudal muscle (Fig. 3c). This flexor runs medial to the adductor caudalis ventralis, which is well developed by this stage, but does not insert onto the fin rays, yet. At stage 5.5 mm SL, new muscles appear via rearrangements of the previous ones. Thus, the flexor caudalis ventralis splits into the large flexor caudalis ventralis superior and small flexor caudalis ventralis inferior, which inserts onto one ventral ray (Fig. 3d). The dorsal caudal muscle breaks up into the flexor caudalis dorsalis superioris and lateralis profundus dorsalis overlying the former. The lateralis profundus ventralis runs closer to the fin rays. During the growth of the tail, the ventral caudal muscle splits into superficial and deep fibers (at 5.6 mm SL), which shift towards the midline. Lastly, the amount of superficial fibers becomes reduced, while deep fibers increase in number, and now instead of inserting onto fin rays they insert onto proximal caudal bones and vertebrae (Fig. 4a,b). At 6.4 mm SL, long and very thin fibers of the lateralis superficialis dorsalis appear (Fig. 4a), and the interradialis caudalis already connects the bases of the all three long dorsal rays (Fig. 4b). The last muscles to develop are the lateralis superficialis ventralis, interfilamenti dorsalis, and interfilamenti ventralis. The three muscles can be seen at 6.7 mm SL (Fig. 4c). At this stage, basically all muscles are present and have a configuration that resembles the adult one^10^. Interestingly, in addition to these muscles, in young specimens the space between the hypurals was filled with muscles (Fig. 5, Supplementary Fig. S3) that disappear in adult fishes when this space becomes smaller. These muscle fibers can be distinguished by their elongated shape, which contrasts with the rhomboid/quadrangular cells of hypurals. We observed these muscle fibers between hypurals 1–2, 2–3 and 3–4 from 5.00 mm SL to 7.1 mm SL, forming very thin muscles that we designate here as *interhypurales*.

### Pectoral fins

**Figure 6:**
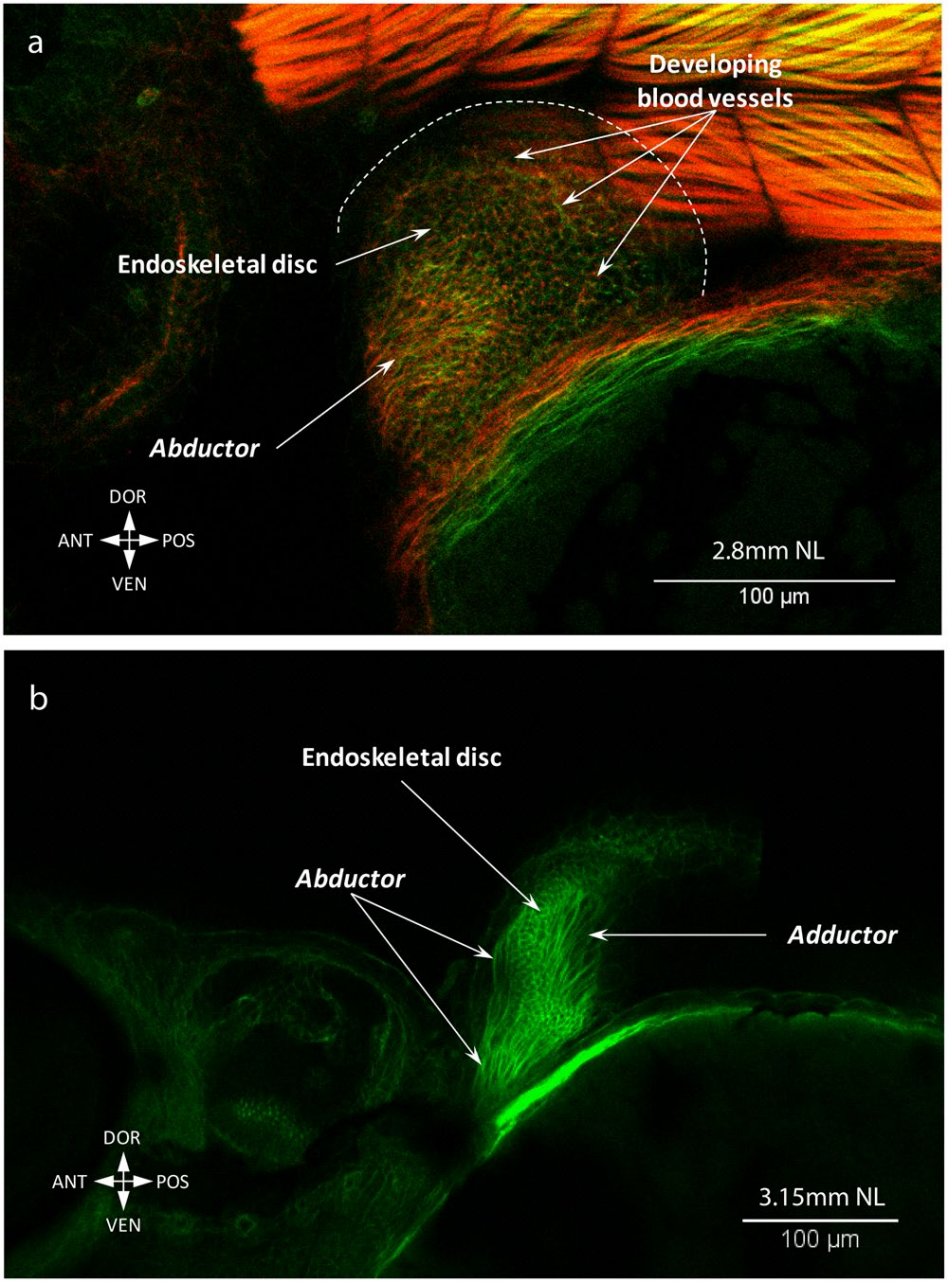
Early development of the pectoral fin muscles. Myofibrils of the abductor and adductor are already present at late embryonic stages, 2.8 mm SL (46 hpf) (**a**). The abductor and adductor muscles extend along the pectoral fin in freshly hatched larvae, 3.15 mm NL (**b**). Note: different colors in (**a**) do not mean different staining, but were used to better show different layers and contrast myoblasts (single dots) and cells of the endoskeletal disc (nets of actin).

**Figure 7:**
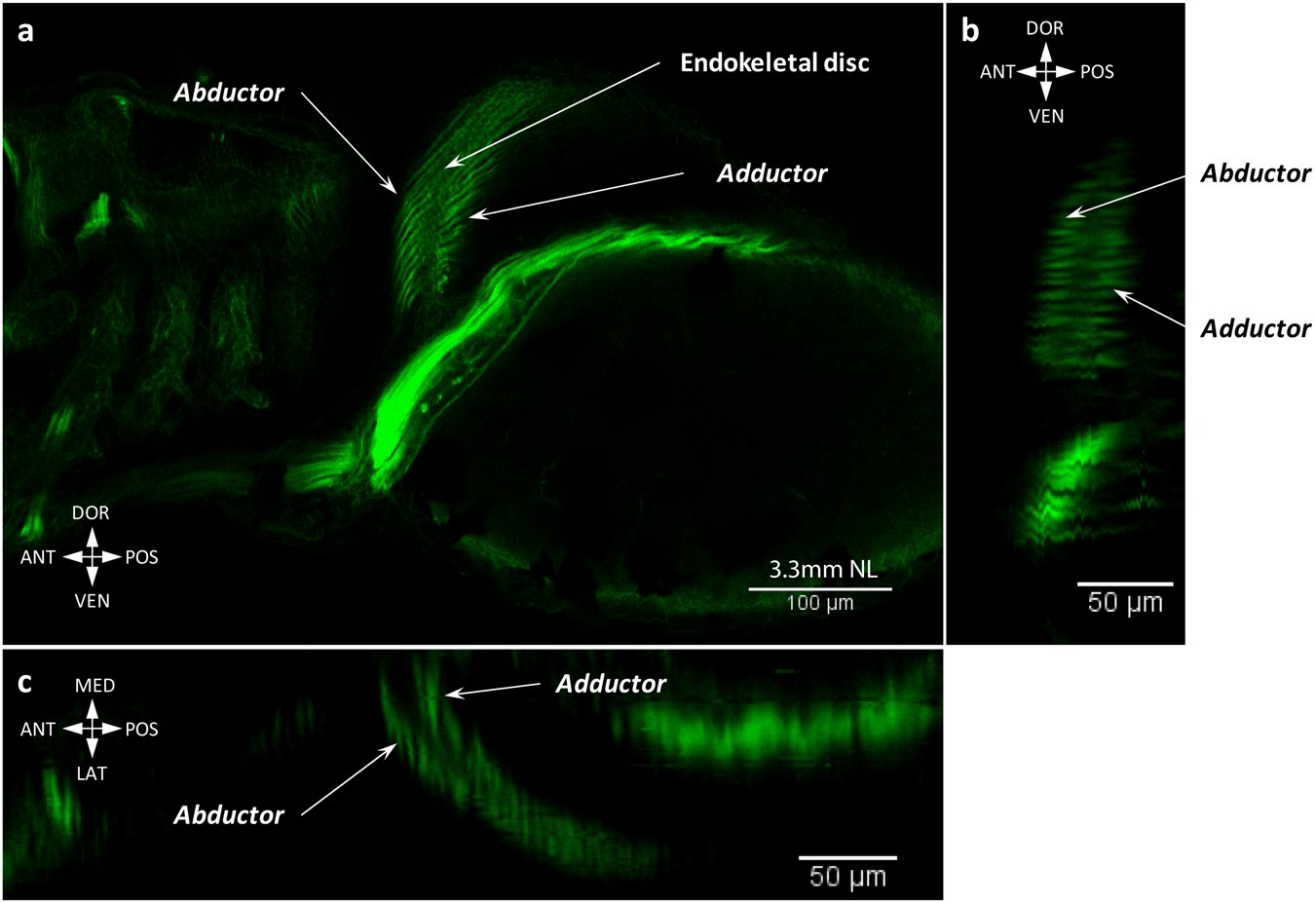
Abductor and adductor form two muscle layers on both sides of the zebrafish pectoral fin. Lateral view (**a**), dorso-ventral (**b**), and anteroposterior (**c**) crossections show that abductor and adductor muscles extend to the edge of the endoskeletal disc and form two muscle layers at 3.3 mm NL.

**Figure 8:**
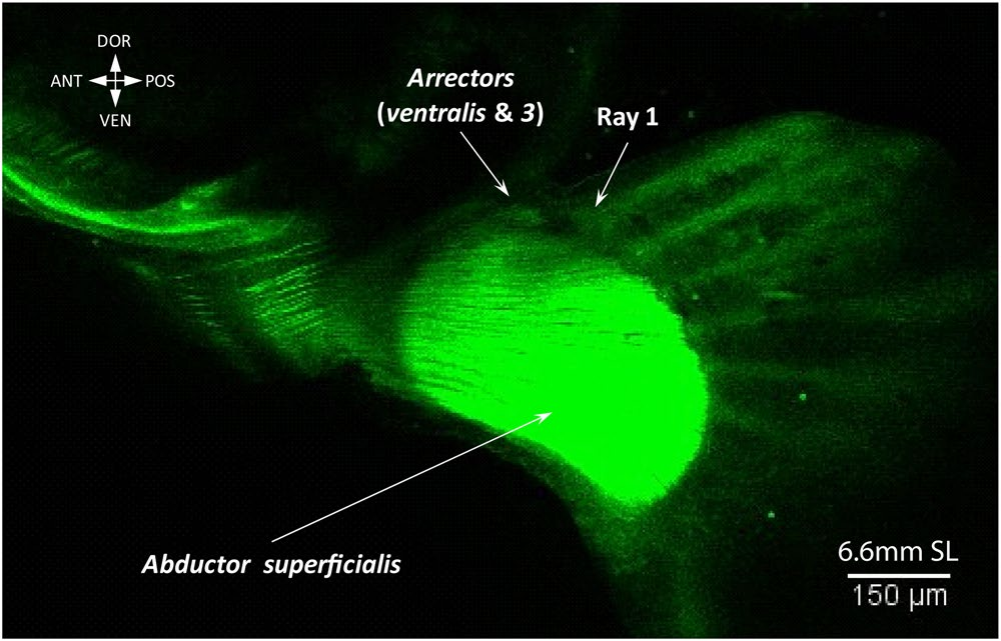
Ventral arrector complex of the pectoral fin in the zebrafish at 6.6 mm SL. The ventral arrector complex will later give rise to the arrector ventralis and arrector-3.

**Figure 9:**
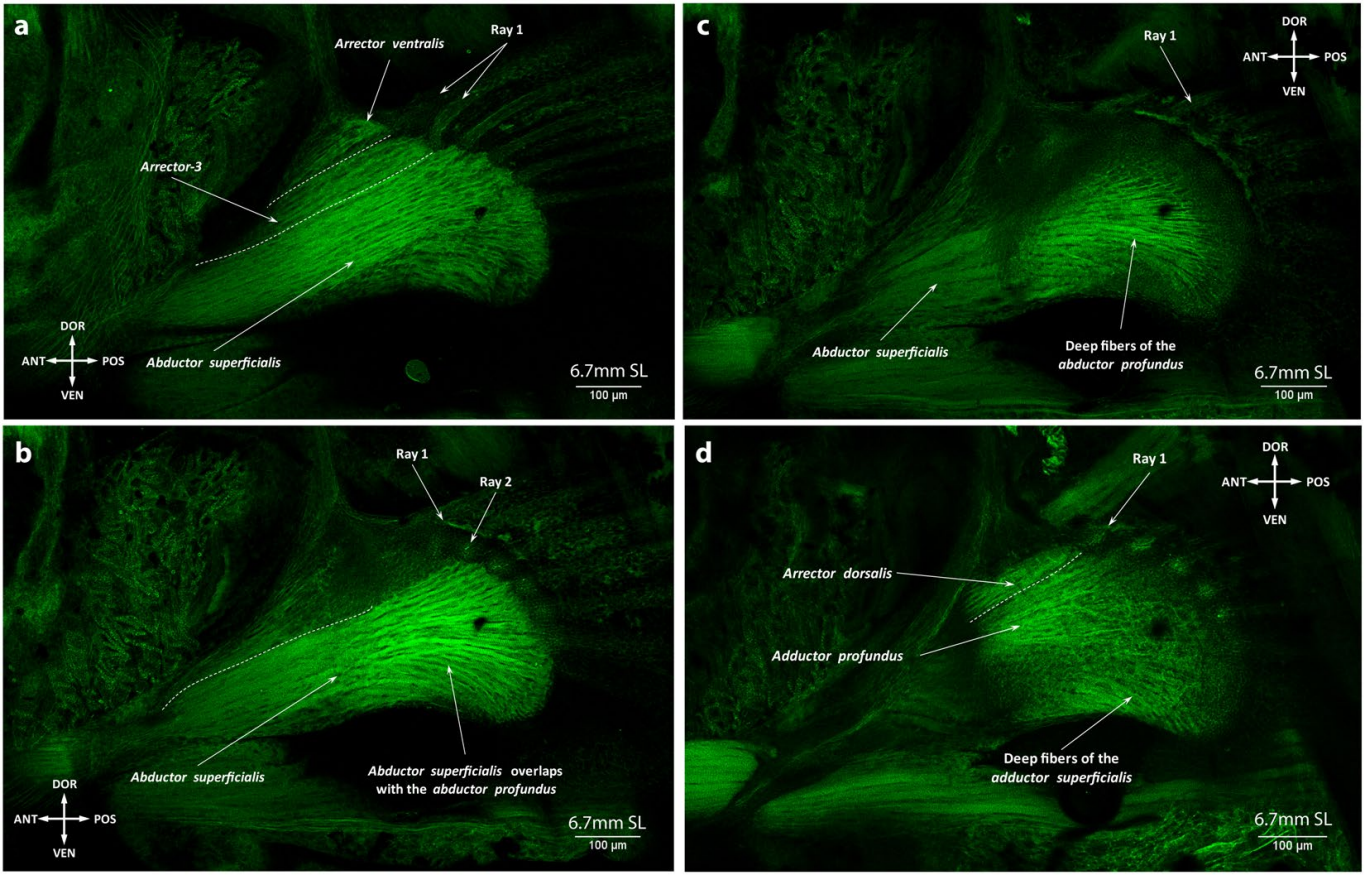
Adult-like configuration of the pectoral fin musculature: all lateral (**a** and **b**) and medial (**c** and **d**) pectoral muscles are developed by 6.7 mm SL.

The pectoral fin buds are already formed by 2.65 mm NL (Fig. 1)^4,20,21^. Our results and previous studies have shown that pectoral fin musculature start developing during early embryogenesis (Fig. 6a)^4,20^ and both abductor and adductor muscle masses differentiate as early as 2.8 mm NL (~46 hours post fertilization) (Fig. 6a)^20^. Before (2.65-2.9 mm NL) and after hatching (3.15 mm NL) of larvae, we observe continuous fibers of the abductor and adductor masses (Fig. 6, Supplementary Fig. S4). By 3.3 mm NL fibers extend to the edge of the endoskeletal disc of the pectoral fin, which has unmineralized actinotrichia (Fig. 7)^20^. Along with fin growth, the abductor and adductor extensively increase in size until 6.4 mm SL, when they split into deep and superficial layers (adductor profundus and superficialis; abductor profundus and superficialis). At 6.6 mm SL, a small bundle attached to the first pectoral ray starts separating from the abductor superficialis (Fig. 8). This bundle later gives rise to both the arrector ventralis and arrector-3 (Fig. 9a). On the medial side only one arrector (arrector dorsalis) develops, apparently from the adductor mass (Fig. 9d). At 6.7 mm SL, all seven pectoral fin muscles are present and display the adult configuration (Fig. 9).

### Dorsal and anal fins

**Figure 10:**
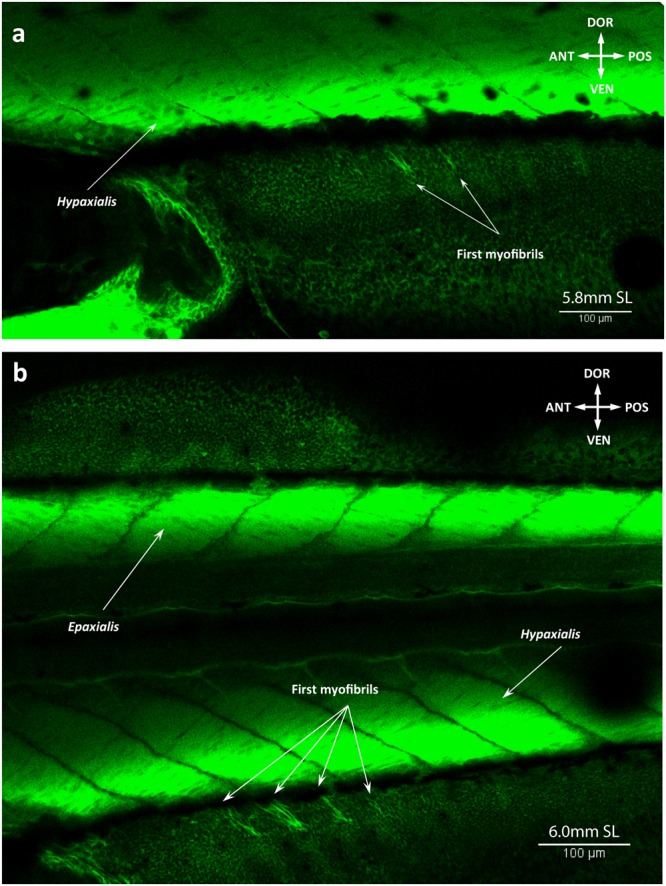
Early development of the dorsal and anal fin musculature in the zebrafish. First muscle fibers are seen in the anal fin at 5.8 mm SL (**a**). Number of fibers and serial units with the muscle fibers increases by 6.0 mm SL (**b**).

**Figure 11:**
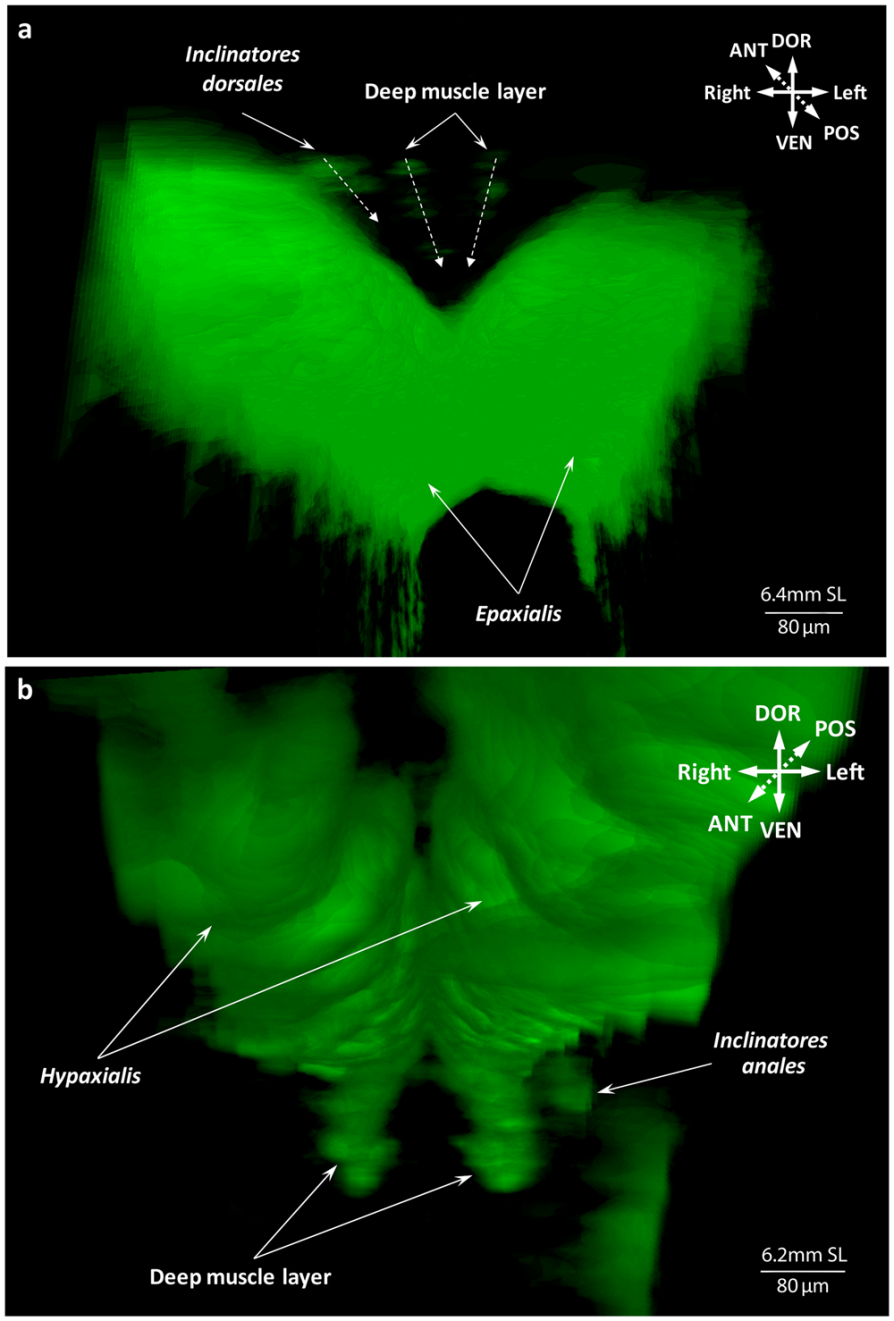
Two muscle layers in the dorsal and anal fins at 6.2 mm SL. The superficial muscle layer of the dorsal (**a**) and anal (**b**) fins corresponds to inclinators. The deep muscle layer will later split into erectors and depressors.

**Figure 12:**
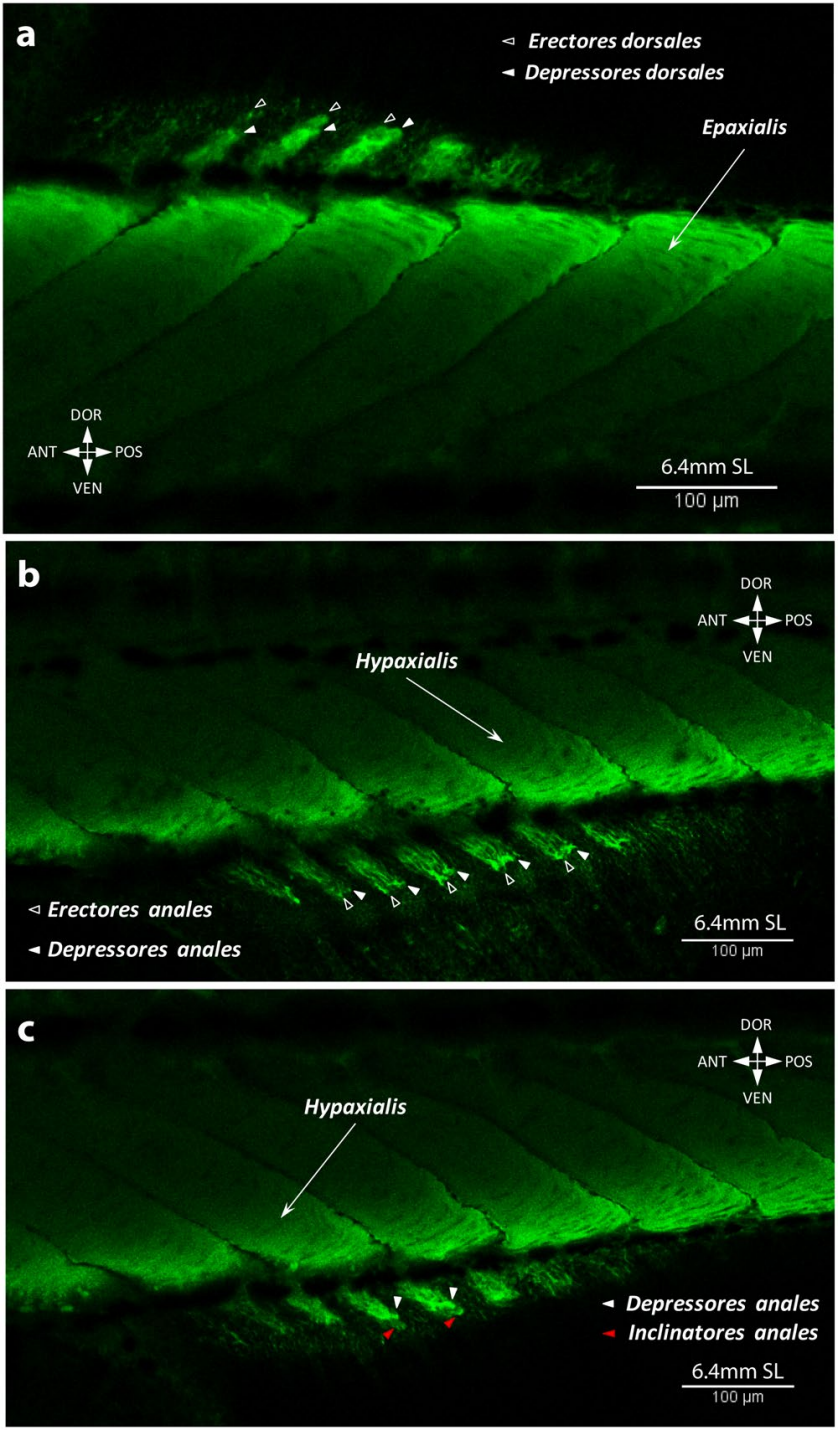
Muscular composition of serial units in the dorsal and anal fins of the zebrafish at 6.4 mm SL. Erectors and depressors of the dorsal (**a**) and anal (**b**) fins form the deep muscle layer, covered with superficial inclinators (**c**) at 6.4 mm SL.

**Figure 13:**
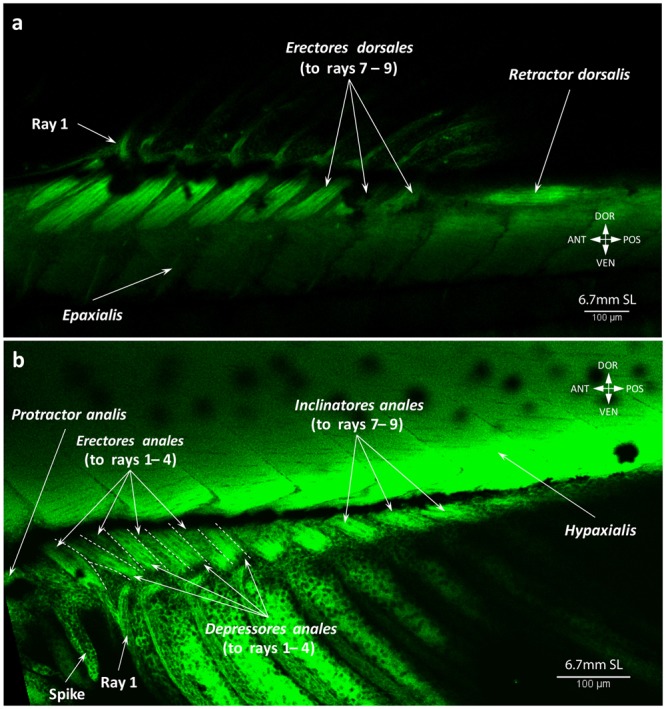
Adult-like configuration of the dorsal and anal fin musculature. Origin of the protractors and retractors in the dorsal (**a**) and anal (**b**) fins at 6.7 mm SL lead to the completion of the development and adult muscle configuration in these fins.

We describe these two fins together because of the striking similarity of their adult anatomy and ontogenetic development. At 5.8 mm SL, muscle fibers appear in the region of several middle rays of the anal fin (Fig. 10a). By 6.0 mm SL, these fibers elongate proximally and distally towards the body and fin rays respectively and myofibrils appear in more anterior and posterior serial units of the fin (Fig. 10b). The dorsal fin musculature develops slightly later than the anal fin musculature and by 6.0 mm SL no muscle fibers can be seen (Fig. 10b). By this stage, any structural rearrangements mainly occur in the same proximo-distal plane, but at 6.2 mm SL muscle differentiation into deep and superficial layers is visible (Fig. 11). The deep layer soon gives rise to the erectors and depressors of each ray (Fig. 12), which are covered by the overlying superficial inclinators (Fig. 12c). Because development of muscles corresponding to different rays is asynchronous, the dorsal and anal fins have muscle units (i.e. including an inclinator, depressor, and erector going to both the left and right sides of each ray, which therefore receives six muscles in total) developed to a different extent at the same stage. Thus, muscle units of the rays that are more central antero-posteriorly can have all six muscles differentiated into superficial-deep layers while the outermost rays may have undifferentiated developing muscle fibers or even single myofibrils (Fig. 12). By 6.7 mm SL, development of each muscle unit is accomplished and the depressors partially overly the erectors, which are subdivided into two small heads attached to the dorsal fin rays (Fig. 13a). In addition to these units of six muscles going to each ray, there are also two longitudinal muscles that develop by 6.7 mm SL within each fin to move the first and last rays, which are named the protractor and retractor analis and the protractor and retractor dorsalis (Fig. 13). Therefore, by 6.7 mm SL, all dorsal and anal fin muscles are already present and have a configuration that resembles that in adults.

### Pelvic fins

**Figure 14:**
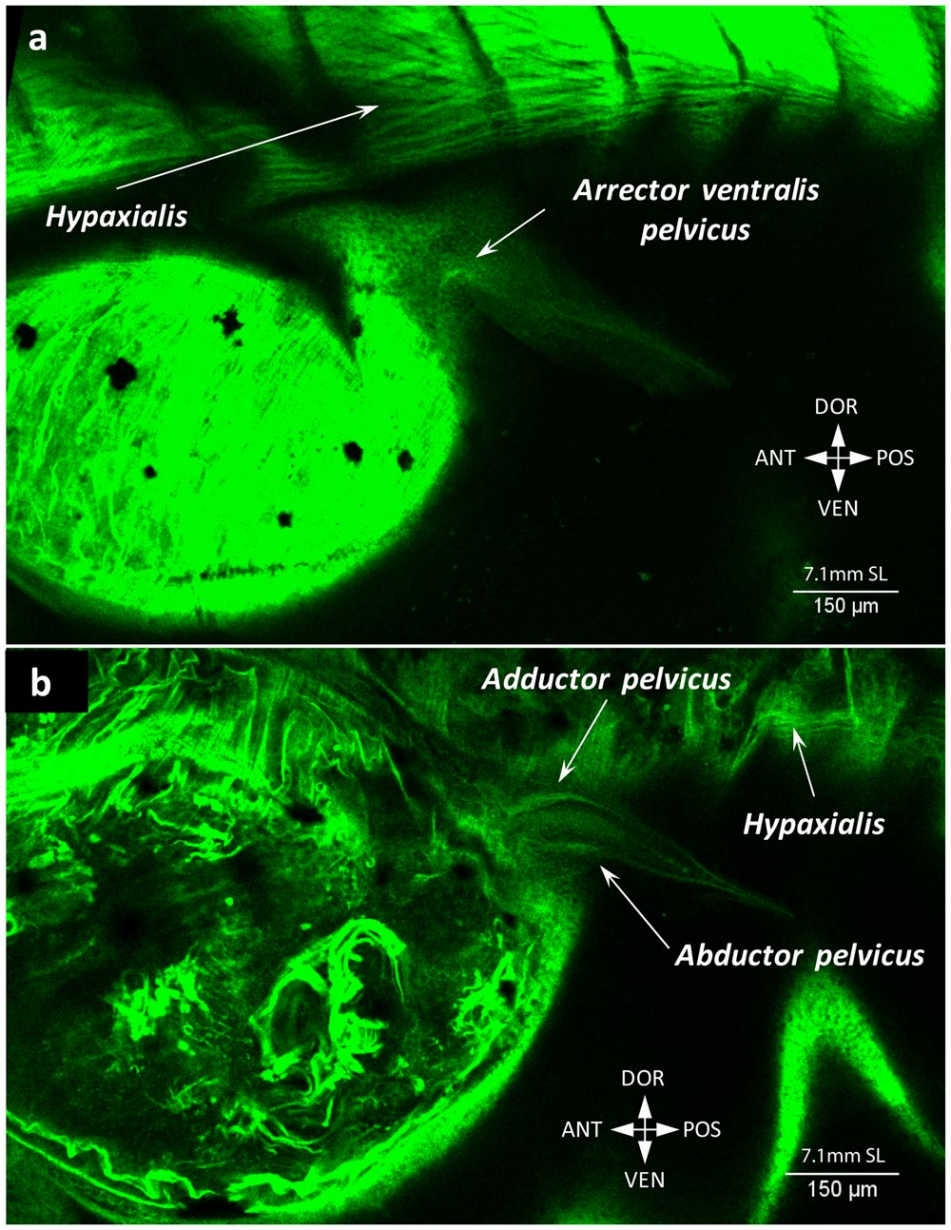
Early development of the pelvic fin musculature in the zebrafish. At 7.1 mm SL, fibers of the initial three muscles are present: arrector ventralis pelvicus (**a**), abductor and adductor pelvicus (**b**). Fibers of the arrector ventralis pelvicus do not reach the fin rays.

**Figure 15:**
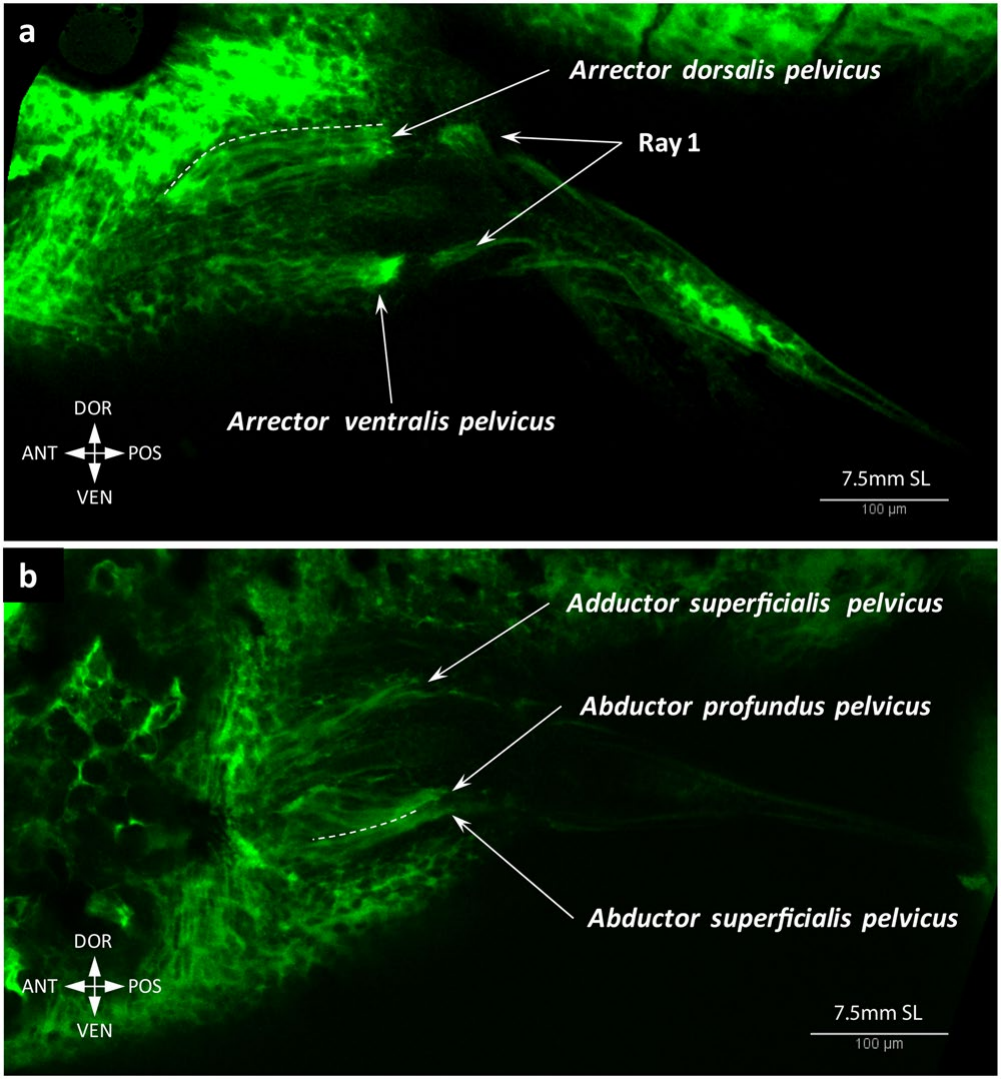
Two arrectors of the pelvic fin are formed by 7.5 mm SL. Both the arrector dorsalis pelvicus and arrector ventralis pelvicus are well developed (**a**). The abductor and adductor muscles start segregating into the deep and superficial layers (**b**).

**Figure 16:**
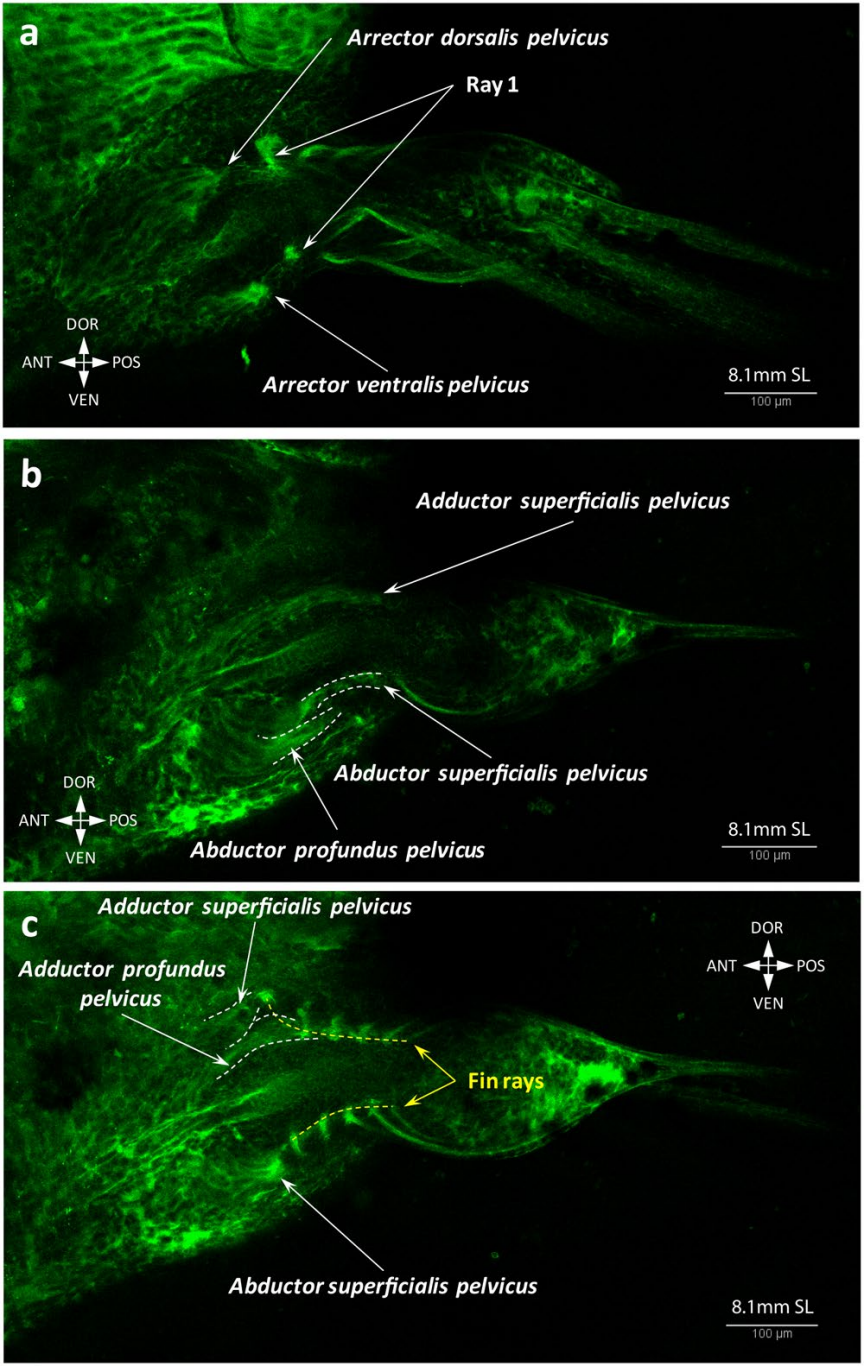
Adult-like configuration of the pelvic fin musculature. Both arrectors (**a**), abductors (**b**) and adductors (**c**) are present at 8.1 mm SL.

Pelvic fins are the last to develop in the zebrafish (Fig. 1). Their buds become visible after 6.7 mm SL. At 7.1 mm SL each pelvic fin already has three differentiated muscle masses (Fig. 14)^17^: the undifferentiated abductor and adductor consist of long thin muscle fibers that stretch proximo-distally along the fin for approximately 1/3 of its length (Fig. 14b), and in addition there is also an arrector ventralis pelvicus (Fig. 14a). Notably, fibers of the arrector dorsalis pelvicus were seen until 7.1 mm SL. The growth of the muscles proceeds quickly and at 7.5 mm SL both arrectors are well developed and attach to the base of the first ray (Fig. 15a, Supplementary Fig. S5). The abductor and adductor muscle masses differentiate into the deep and superficial layers (abductor superficialis and profundus pelvicus; adductor superficialis and profundus pelvicus), which are still difficult to recognize at this stage (Fig. 15b, Supplementary Fig. S5). By 8.1 mm SL, all pelvic muscles are clearly present and have an adult configuration (Fig. 16).

## Discussion

Unlike other fins, which are functionally and developmentally distinct structures locally connected to the body, the caudal fin is in a way mainly a posterior continuation of the trunk, and of the vertebral column in particular. It has been suggested that such a peculiar position and association with the posterior elements of the postcranial axial skeleton make this fin developmentally and evolutionary distinct from other fins^22,23^. Our observations and comparisons provide additional evidence supporting this idea.

From the very early development, the caudal fin is supplied with musculature, while other fins appear as relatively simple homogeneous structures – fin folds or fin buds – that grow and acquire muscles later in development (exceptionally, pectoral fins develop muscles during embryogenesis). In contrast to such gradual development, the caudal fin at 2.95 mm NL already has two muscles (the dorsal and ventral caudal muscles) that differ from the trunk muscles by their orientation and composition (i.e. absence of myomeric pattern) (Fig. 2a). Interestingly, even though the caudal fin is the first fin to appear in the zebrafish, it does not reach an adult-like configuration before other fins do, probably because of its complexity as it includes more muscles and skeletal elements than any other fin (Fig. 1). That is, the caudal fin develops along with the fish growth and the adult muscle configuration of the caudal fin is attained at a similar developmental stage as in the pectoral, dorsal, and anal fins (i.e. by about 6.7 mm SL) (Fig. 4c).

Another distinguishing feature of the caudal fin muscles is the proximal shift during development; i.e., away from the fin rays. While muscles of all other fins mainly grow distally towards the rays and insert onto their bases, the position of the ventral caudal muscle changes from more dorsal to more medial and later this muscle loses the connection to the caudal fin rays (Figs 2c and 3a). In adult fishes, the ventral caudal muscle becomes a deep trunk muscle, attached to the caudal vertebrae and proximal caudal bones. This particular muscle rearrangement along with the intensive growth of other ventral caudal muscles and notochord bending (i.e. the adductor caudalis ventralis and flexor caudalis ventralis superior and inferior) result into the peculiar marked dorsoventral asymmetry of the caudal fin, which initially was symmetrical (until 4.4 mm NL: Fig. 2a). The presence of the temporary interhypural muscles on the dorsal side only, development of the lateralis superficialis dorsalis before the lateralis superficialis ventralis, and appearance of the interradialis dorsalis at 6.4 mm SL enhance the marked difference between the dorsal and ventral sides of this fin. Thus, what appears (externally) to be a dorsoventral symmetrical caudal fin, with roughly equal dorsal and ventral lobes and somewhat evenly distributed fin rays, is a fin with muscles that display a striking dorsoventral asymmetry^8,10^. This dorsoventral asymmetry of the caudal fin is also present in the bone architecture^24^ as well as in the development of rays^19^.

In fact, it is interesting to note that even dorsal and ventral caudal muscles that are relatively symmetric in adults, and are thence commonly described under similar names, display very different developmental patterns. For instance, the lateralis profundus dorsalis, which basically corresponds to the dorsal caudal muscle, is well developed at very early stages (at 3.3 mm SL) and extends to the very tip of the tail (Fig. 2a). In contrast, the lateralis profundus ventralis develops later - first fibers are seen at 4.4 mm SL - and derives from the hypaxial, segmented musculature, not from the ventral caudal muscle (Fig. 2b,c). A similar discrepancy is also seen in the development of the dorsal and ventral flexors. On the ventral side, a single flexor caudalis ventralis bifurcates into the flexor caudalis ventralis superior and flexor caudalis ventralis inferior. In contrast, the flexor caudalis dorsalis superioris and flexor caudalis dorsalis inferioris appear at different stages from different sources: from the dorsal and ventral caudal muscles, respectively (Fig. 2b,c).

In general, the major phylogenetic changes leading, in fishes, from a homocercal tail to a heterocercal tail can be traced in the ontogenetic development of the zebrafish. The asymmetrical heterocercal tail and the notochord extending beyond the most posterior endoskeletal element, seen in most adult non-teleost ray-finned fishes, represents the ancestral state^25^. Unlike Chondrichthyes and Chondrostei, adult gars and bowfins (the closest extant relatives of teleosts) have an externally symmetrical caudal fin. Nevertheless, these fishes are said to have a heterocercal caudal fin as well^25^. The main phylogenetic trend observed across these taxa is the primary development of the ventral caudal musculature; e.g. in sharks, sturgeons, paddlefish^26^. Development of the hypural bones provided an additional support for the ventral part of the caudal fin and made it possible to develop the muscle adductor caudalis ventralis (present in teleosts and in non-teleost fishes as e.g. *Lepisosteus*^26,27^), which has likely changed the unsteady locomotion and acceleration speeds^27^ and contributed to the rapid radiation of teleosts during the Mesozoic^28,29^. The evolution of the ventral caudal musculature was followed by the compensatory development of the dorsal caudal muscles (seen in teleosts and in non-teleost fishes as e.g. bowfins^26,27^). A similar pattern of development of caudal muscles was also observed in the present work during the development of the caudal fin musculature of the zebrafish, which, in adulthood, is a representative of a true homocercal tail.

**Figure 17:**
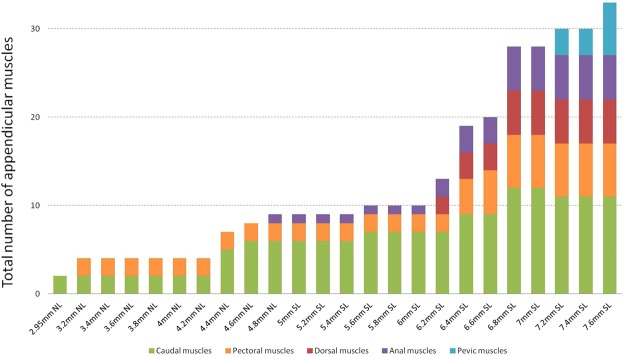
Total number of appendicular muscles during the zebrafish development.

As shown in Fig. 17, the development of the zebrafish appendicular muscles does not proceed at a constant rate during embryonic development: there are clear cases of developmental acceleration and steady states in-between them. At 3.2 mm NL, zebrafish embryos acquire first four appendicular muscle masses: two caudal ones (the dorsal and ventral caudal muscles) and two pectoral ones (the abductor and adductor muscles). From this stage starts the first steady condition that lasts for 1 mm NL change, until 4.2 mm NL, in which these four muscles grow along with the overall size of the tail and pectoral fins. During this period, no new muscles arise. At 4.2 mm NL, the first small developmental burst starts and by 4.8 mm NL five new muscles appear. The first muscle fibers of the anal fin appear at the end of this acceleration time (at 4.8 mm NL), and during the next steady period (4.8 mm NL − 6.0 mm SL) the caudal, pectoral, and anal fins keep a constant number of muscles. The second developmental burst is initiated by the muscle development in the dorsal fin at 6.2 mm SL and leads to the adult-like muscle configuration in all five fin types. During the next 1.4 mm SL change, the number of muscles increases from 13 to 32. The pelvic musculature development starts without passing through a steady state and thus falls within the last third of the second developmental burst.

Some stages of development of the caudal musculature in the zebrafish display some resemblance with the caudal fin configuration seen in adult stages of other fishes and also of non-vertebrate chordates such as the amphioxus (Amphioxiformes). For instance, the dorsal and ventral caudal muscles of the zebrafish are in a certain sense a continuation of the trunk musculature, as noted above. Even though fibers of these two muscles are (secondarily) not segmented into myomeres (Fig. 2a), they are inextricably linked to the trunk muscles and articulate the tail of the animal, which lacks a proper caudal fin at this stage but has a caudal fin fold similar to that of the adult amphioxus^30^. The first caudal muscle (radialis) is said to have evolved in at least the last common ancestor (LCA) of bony fishes and cartilaginous fishes, being for instance present in extant adult sharks^31^, located ventral to the notochord. Accordingly, development of the intrinsic caudal fin in the zebrafish stars on the ventral side of the fin (Fig. 2b,c, Supplementary Fig. S1).

In adult gars (e.g. *Lepisosteus oculatus*), the interradialis hypaxialis (flexor ventralis *sensu* Lauder^27^) and hypochordal longitudinalis are situated below the notochord^27^. A similar muscle configuration is present in the zebrafish at 4.4 mm NL – 5.0 mm SL (Figs 1b,c and 16a). In this case, the interradialis hypaxialis of adult gars (and even phylogenetically more basal actinoperygians such as *Polypterus*) and the radialis of sharks would topologically correspond to the flexor caudalis ventralis (inferior & superior) of early zebrafish stages. The hypochordal longitudinalis would thus topologically correspond to the hypochordalis longitudinalis of adult fishes such as those of the species *Lepomis macrochirus*^32^ and to the adductor caudalis ventralis of the zebrafish^10^. It has been suggested that the hypochordal longitudinalis is evolutionarily derived from the dorsal portion of the interradialis hypaxialis^27^ but the present study indicates that the analogous adductor caudalis ventralis of the zebrafish does not develop from the flexor caudalis ventralis (Fig. 2b). It is worth looking in more detail and investigating whether the hypochordal longitudinalis might have had an evolutionary origin different from the one proposed by Lauder^27^. The potential candidate for such an evolutionary origin could be the ventral caudal muscle, which in zebrafish development overlies the early adductor caudalis ventralis and thus could be a source of the first adductor fibers. Further studies are thus needed in order to test each of the three following hypothesis and to clarify whether the hypochordal longitudinalis (and the analogous adductor caudalis ventralis) was evolutionarily: (1) derived from the interradialis hypaxialis as proposed by Lauder^27^, i.e. that both these muscles are somewhat homologous to the radialis of adult sharks and chondrosteans; (2) derived from the deep fibers of the ventral caudal muscle that became attached to the posterior caudal vertebrae (e.g. as seen in adult *Amia*) instead of the hypaxialis; or (3) a *de novo* intrinsic caudal muscle acquired during the origin of the Neopterygii.

Dorsal caudal fin muscles appear later in phylogeny, e.g. the interradialis dorsalis and supracarinalis are found in adult stages of teleosts and of non-teleostean fishes such as *Amia* but not of more phylogenetically basal taxa such as *Polypterus* and chondrosteans^27,32^. In zebrafish development the development of the dorsal flexors and the specification of the lateralis profundus dorsalis occurs at 5.2–5.5 mm SL and the interradialis caudalis appears at 6.4 mm SL. According to Lauder^27^ the interradialis muscles (analogous to the complex of the interradialis caudalis and interfilamenti caudalis dorsalis and ventralis in the zebrafish) arose evolutionarily in two steps: first the interradialis caudalis dorsalis evolved, as seen in e.g. adults of *Amia*^27^ but not of *Polypterus* and chondrosteans, and then the interradialis caudalis ventralis evolved, as seen in adults of many extant teleostean taxa^27,32^. In zebrafish the development the interradialis caudalis precedes that of the interfilamenti caudalis. Nevertheless, the interfilamenti caudalis dorsalis and interfilamenti caudalis ventralis develop simultaneously. The absence of the expected temporary delay (dorsal vs ventral muscles) could be related to the accelerated development and rapid changes in the overall zebrafish morphology at 6.4–6.7 mm SL, which fall into the second developmental burst of Fig. 17 (see Fig. 4). In particular, in the zebrafish both the interradialis caudalis dorsalis and interradialis caudalis ventralis develop within the frame of a 0.3 mm SL change: at 6.4 mm SL we could only see the dorsal interradialis caudalis, while at 6.7 mm SL all muscles between the caudal fin rays were fully developed (Fig. 17).

Thus, general trends of muscle development in the zebrafish caudal fin are the following: the primary development of the ventral caudal musculature, followed by the development of the dorsal caudal muscles; development of the dorsal between-ray musculature precedes the ventral one. As noted above, the presence of hypural bones probably provided an additional support for the ventral part of the caudal fin and could have facilitated the development of the adductor caudalis ventralis, which might well have effected the unsteady locomotion and acceleration speeds^27^ and contributed to the rapid radiation of teleosts during the Mesozoic^28,29^.

Regarding the pectoral fin musculature, at early stages of zebrafish development it comprises two muscle masses attached to the endoskeletal disc – the abductor and adductor masses (Figs 5 and 6)^9^. Such simple muscle composition has been hypothesized to represent the plesiomorphic state for the pectoral appendages^7,33^. Further differentiation of muscles from the abductor mass going to the first ray in the zebrafish^9^ (the arrector ventralis) was likely acquired in the last common ancestor of extant gnathostomes^33^. Development of the arrector-3 occurred later, during teleost evolutionary history. Similarly, the separation of the arrector-3 from the arrector ventralis complex is the last major change occurring during the development of the zebrafish pectoral fin.

Concerning the pelvic appendage, the first three muscular structures to evolve in fishes were the adductor and abductor masses and the arrector ventralis pelvicus, all of them being very likely present in the LCA of extant gnathostomes^33^. The arrector dorsalis was apparently acquired during the evolutionary history of actinopterygians, because it is absent in chondrichthyans, sarcopterygians and the phylogenetically basal actinopterygian *Polypterus*^33,34^. The present work shows that in zebrafish development the arrector dorsalis develops later than the other muscles of the pelvic fins, as it did evolve later than them in evolution.

Unfortunately, muscles of the anal and dorsal fins have not been well described in the literature, particularly for non-teleostean fishes (for a recent discussion on such lack of comparative data, see Siomava and Diogo^10^). Therefore it is difficult to compare the development of these muscles in the zebrafish with the adult configurations found in other, non-teleostean fishes. Hopefully, the present study will stimulate and pave the way for future developmental, comparative, evolutionary and evo-devo studies of the appendicular muscles, and in particular of the ones of the median fins, in fishes. Such studies are crucially important to clarify the origin, development and evolution of the appendages of gnathostomes as well the fins-limbs transitions during the origin of tetrapods.

## Electronic supplementary material

Dataset 1
