# Supplementary material for: Development of zebrafish paired and median fin musculature: basis for comparative, developmental, and macroevolutionary studies
Source: Sci Rep. 2018 Sep 21;8:14187. doi: 10.1038/s41598-018-32567-z (PMC6155031; doi:10.1038/s41598-018-32567-z)
Supplement: Supplementary file 1 — Dataset 1 [file 41598_2018_32567_MOESM1_ESM.zip › Supplementary Figure Legends.pdf]

Supplementary Fig. S1 Early development of the ventral caudal muscles, stage 4.4mm NL.

Supplementary Fig. S2 Hypyral complex and caudal muscles at stage 4.6 mm NL.

Supplementary Fig. S3 Deep interhypural fibers in the caudal fin of *Danio rerio*, 6.0mm SL

Supplementary Fig. S4 The abductor and adductor muscles of the pectoral fin at stage 3.15mm NL. Muscle fibers extend along the pectoral fin in freshly hatched larvae and form two layers.

Supplementary Fig. S5 Musculature of the pelvic fin at stage 7.5mm SL.
