# Supplementary figures and images for: Development of zebrafish paired and median fin musculature: basis for comparative, developmental, and macroevolutionary studies
Source: Sci Rep. 2018 Sep 21;8:14187. doi: 10.1038/s41598-018-32567-z (PMC6155031; doi:10.1038/s41598-018-32567-z)

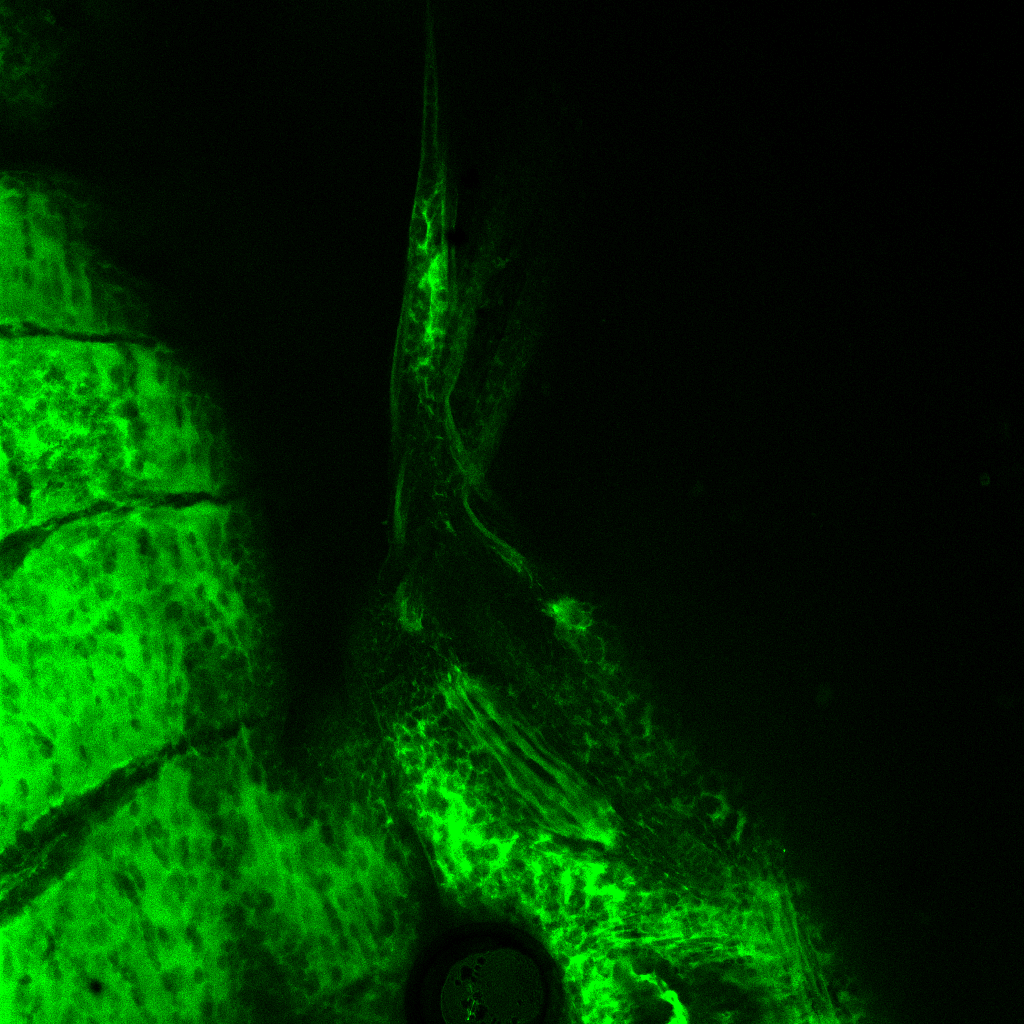

Supplement: Supplementary file 1 — Dataset 1 [file 41598_2018_32567_MOESM1_ESM.zip › Fig. S5.gif]

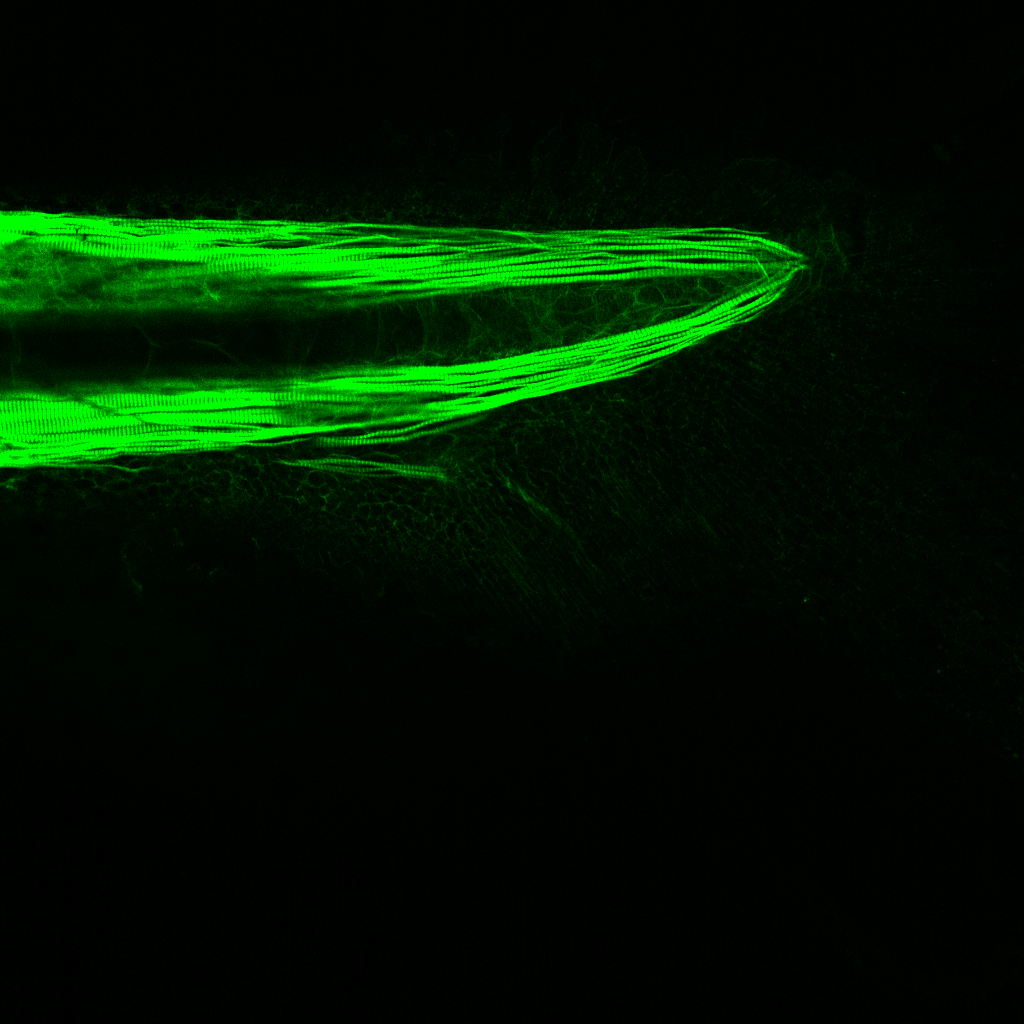

Supplement: Supplementary file 1 — Dataset 1 [file 41598_2018_32567_MOESM1_ESM.zip › Fig. S1.gif]

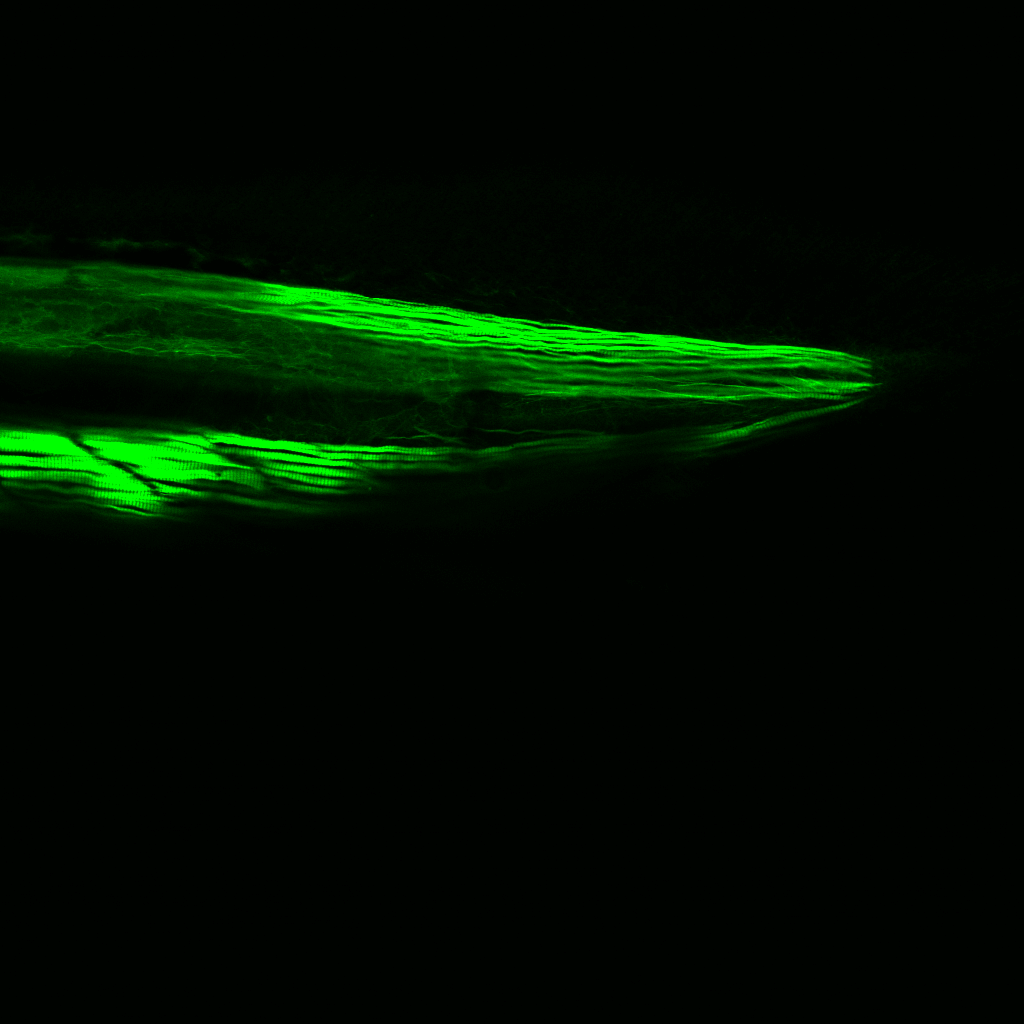

Supplement: Supplementary file 1 — Dataset 1 [file 41598_2018_32567_MOESM1_ESM.zip › Fig. S2.gif]

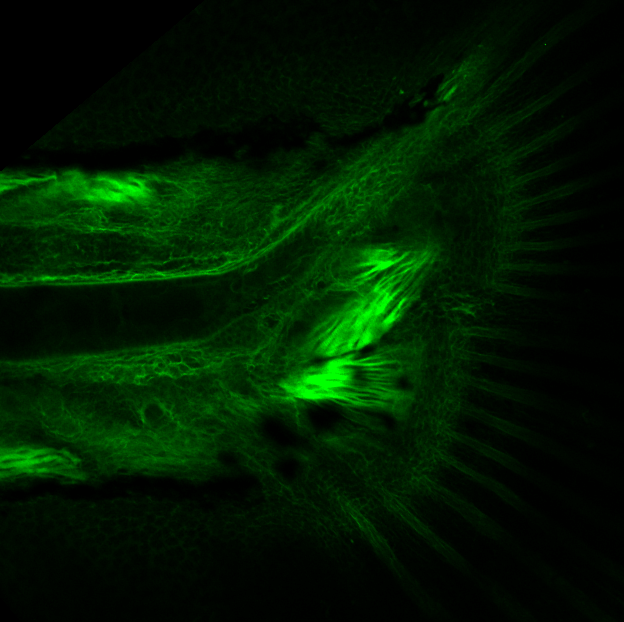

Supplement: Supplementary file 1 — Dataset 1 [file 41598_2018_32567_MOESM1_ESM.zip › Fig. S3.gif]

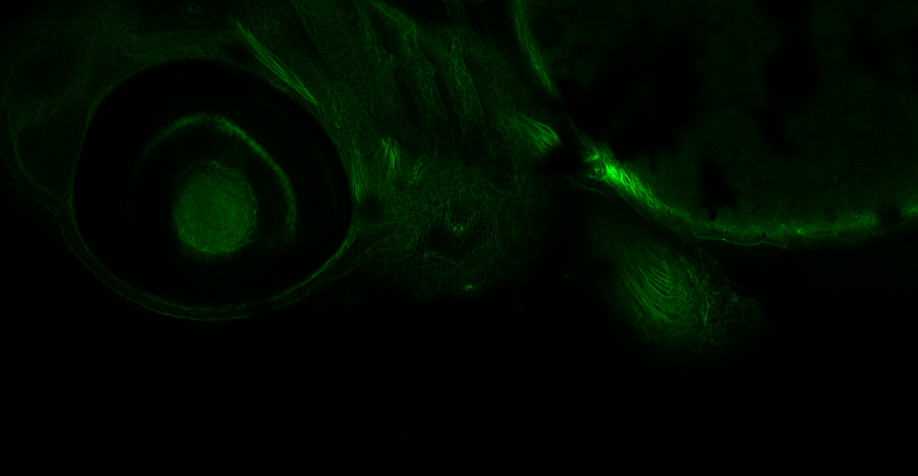

Supplement: Supplementary file 1 — Dataset 1 [file 41598_2018_32567_MOESM1_ESM.zip › Fig. S4.gif]
